# Functional Enhancement of AT1R Potency in the Presence of the TPαR Is Revealed by a Comprehensive 7TM Receptor Co-Expression Screen

**DOI:** 10.1371/journal.pone.0058890

**Published:** 2013-03-14

**Authors:** Jonas Tind Hansen, Christina Lyngsø, Tobias Speerschneider, Pernille B. L. Hansen, Céline Galés, David M. Weiner, Søren P. Sheikh, Ethan S. Burstein, Jakob Lerche Hansen

**Affiliations:** 1 Laboratory for Molecular Cardiology, Department of Biomedical Sciences and The Danish National Research Foundation Centre for Cardiac Arrhythmia, Faculty of Health Sciences, University of Copenhagen, Copenhagen, Denmark; 2 Department of Clinical Biochemistry, Glostrup Hospital, Glostrup, Denmark; 3 Cardiovascular and Renal Research, University of Southern Denmark, Odense C, Denmark; 4 Institut des Maladies Métaboliques et Cardiovasculaires (I2MC), Institut National de la Santé et de la Recherche Médicale, Université Toulouse III Paul Sabatier, Toulouse, France; 5 Proteostasis Therapeutics, Inc., Cambridge, Massachusetts, United States of America; 6 Department of Clinical Biochemistry and Pharmacology, Laboratory for Molecular and Cellular Cardiology, Odense University Hospital, Odense, Denmark; 7 ACADIA Pharmaceuticals, Inc., San Diego, California, United States of America; 8 Diabetes NBEs and Obesity biology, Novo Nordisk, Måløv, Denmark; Medical School of Hannover, United States of America

## Abstract

**Background:**

Functional cross-talk between seven transmembrane (7TM) receptors can dramatically alter their pharmacological properties, both *in vitro* and *in vivo*. This represents an opportunity for the development of novel therapeutics that potentially target more specific biological effects while causing fewer adverse events. Although several studies convincingly have established the existence of 7TM receptor cross-talk, little is known about the frequencey and biological significance of this phenomenon.

**Methodology/Principal Findings:**

To evaluate the extent of synergism in 7TM receptor signaling, we took a comprehensive approach and co-expressed 123 different 7TM receptors together with the angiotensin II type 1 receptor (AT1R) and analyzed how each receptor affected the angiotensin II (AngII) response. To monitor the effect we used integrative receptor activation/signaling assay called Receptor Selection and Amplification Technology (R-SAT). In this screen the thromboxane A2α receptor (TPαR) was the only receptor which significantly enhanced the AngII-mediated response. The TPαR-mediated enhancement of AngII signaling was significantly reduced when a signaling deficient receptor mutant (TPαR R130V) was co-expressed instead of the wild-type TPαR, and was completely blocked both by TPαR antagonists and COX inhibitors inhibiting formation of thromboxane A_2_ (TXA_2_).

**Conclusions/Significance:**

We found a functional enhancement of AT1R only when co-expressed with TPαR, but not with 122 other 7TM receptors. In addition, the TPαR must be functionally active, indicating the AT1R enhancement is mediated by a paracrine mechanism. Since we only found one receptor enhancing AT1R potency, our results suggest that functional augmentation through 7TM receptor cross-talk is a rare event that may require specific conditions to occur.

## Introduction

The angiotensin II type 1 receptor (AT1R) belongs to the super-family of seven-transmembrane (7TM) or G protein coupled receptors (GPCRs). AT1R is a key regulator of blood pressure and salt and water homeostasis in the Renin-Angiotensin System (RAS). The receptor is implicated in renal and cardiovascular pathophysiology and modern drug therapy involves the use of AT1R blockers and inhibitors of the angiotensin-converting enzyme [1], [2], [3].

During the last two decades the concept of what constitutes a functional entity of 7TM receptors has evolved from a simplistic one receptor:one G protein system. Several studies show that receptors can cooperate either through physical interaction as dimers or higher order oligomers, or by employing functional cross-talk between non-attached receptors [4], [5], [6], [7]. The interplay between particular receptors can modify the response from either or both/all receptors to stimuli encountered by the cell. This may have implications for drug development by allowing the design of drugs that target specific sub-populations of receptors [8].

For the AT1R several examples of both homo- and heterodimers as well as functional cross-talk have been reported. AT1R homo-dimerization has been shown in a number of studies [9], [10], [11]. Regarding heterodimers, it has been shown that the AT1R decreases Gα_q_ coupling when the receptor interacts with either Ang(1–7) receptor (MAS) or angiotensin II type 2 (AT2) receptor [12], [13], [14], [15] and AT2R cross-inhibits AT1R internalization [16]. Additionally, the AT1R has been shown to form complexes with the β2-adrenergic receptor [17], physically interact with the apelin receptor [18], and form heterodimers with α_1D_ adrenoceptor during pregnancy-induced hypertension [19]. The AT1R was also proposed to form heterodimers with the Bradykinin B2 receptor [20], but this finding has failed to be reproduced in several other laboratories [21], [22].

Modification of signal transduction cascades also occurs between receptors that do not physically interact as a consequence of paracrine mechanisms. This was elegantly shown by Turu et al. in which the CB_1_ cannabinoid receptor was activated by the AT1R through a paracrine transactivation mechanism [5]. In addition, dopamine D1/D3/D5 receptors may also modify AT1R signaling, but the mechanism underlying these effects remains to be determined [23], [24], [25].

To investigate how widespread 7TM receptor cross-talk actually is, we utilized a high-throughput system called Receptor Selection and Amplification Technology (R-SAT) [27], [31]. Previously we have shown that R-SAT is effective in detecting functional interactions between receptors and that it also allows for large scale screening [21], [26], [27]. In this study, we used R-SAT in combination with other techniques to analyze how co-expression of 123 individual 7TM receptors influenced the signaling properties of the AT1R.

## Materials and Methods

### Materials

Angiotensin II (A9525) and 9,11-Dideoxy-11α,9α-epoxymethanoprostaglandin F2α (U46619) (D8174) were purchased from Sigma Aldrich, SQ29548 (19025) was purchased from Cayman Chemical, *myo*-[2-^3^H]inositol was purchased from Amersham Biosciences, Coelenterazine 400a (DeepBlueC™) (C-7011) and Coelenterazine h (C-7004) were purchased from Biosynth.

### Recombinant DNA Plasmids

The enhanced GFP-tagged bovine β-arrestin2 plasmid and AT1R-*R*luc plasmid constructs were described previously as well as the rAT1aR and the hAT1R-pSI plasmids [9], [28]. Plasmids encoding Gβ1, GFP10-Gγ_2_, Gα-*R*luc8, TPαR, and TPαR-*R*luc were previously reported [29], [30]. The sequence of all constructs was verified by sequencing.

### Cell Culture and Transfection

Human embryonic kidney 293 cells (HEK 293) and COS-7 cells were maintained in Dulbecco’s modified Eagle medium (DMEM) Glutamax supplemented with 10% (v/v) foetal bovine serum (FBS), and 100 units/mL-1 penicillin/streptomycin at 37°C in 5% CO_2_ atmosphere.

Transient transfection were performed 24 hours after cell seeding with Polyethylenimine (PEI, Polysciences Inc.) or Lipofectamine2000 (Invitrogen) according to manufacturer’s protocol. Within experiments the total concentration of DNA was kept constant by adding appropriate amount of vector pCDNA3.1 plasmid.

### Receptor Selection and Amplification Technology (R-SAT)

The R-SAT assay utilizes the growth characteristics of NIH3T3 cells. Normally, NIH3T3 cells become contact inhibited upon reaching confluency. Transiently expressed oncogenes, proto-oncogenes, and many 7TM receptors confer partial or total transformation of these cells, causing a loss of contact inhibition and allowing them to continue to proliferate beyond this point [27], [31]. In R-SAT, a reporter gene (in this case β-galactosidase) is co-transfected with the 7TM receptor of interest. The β-galactosidase reporter is constitutively expressed and does not participate in driving the biological response, but rather works as an indirect quantitative measure of proliferation [27]. The R-SAT assay was performed as previously described [28], [32]. Briefly, NIH3T3 cells at 70 to 80% confluence in DMEM supplemented with 10% FBS, penicillin (100 U/ml), streptomycin (100 g/ml) were transfected with human AT1R cDNA alone or in combination with the 7TM receptor of interest (5 ng of receptor/well and 20 ng of β-galactosidase reporter/well of a 96-well plate) using the PolyFect Reagent (QIAGEN, Valencia, CA) as described in the manufacturer’s protocol. One day after transfection, ligands were added in DMEM supplemented with 10% FBS, penicillin (100 U/ml), streptomycin (100 g/ml), and 2% Cyto-SF3. After 6 days, the media was aspirated and cells lysed. O-nitrophenyl-β-D-galactopyranoside was added, and the resulting absorbance was measured spectrophotometrically. All concentration–response curves were performed in duplicate.

### IP Accumulation

4.0 million HEK293 cells were seeded into a p10 dish and grown in DMEM supplemented with 10% FBS, penicillin (50 units/mL), streptomycin (50 units/mL) and glutamine (2 mM). After 24 h, the cells were transfected using PEI. The next day cells were split into poly-D-lysine coated 96-well plates (50.000 cells/well) in inositol-free DMEM supplemented with non-essential amino acids, 10% FCS and myo-[2-^3^H]inositol (2 µL/mL medium) (Amersham Biosciences). Cells were stimulated with increasing concentrations of ligands for 20 minutes at 37°C. Ligands were removed, and cells were incubated on ice with formic acid (10 mM) for at least 45 min. 20 µL of the lysis solution was transferred to a solid white 96-well plate, and 80 µL of freshly diluted SPA YSi beads (12 mg/mL) were added. The plates were shaken vigorously on a shaker for half an hour, and incubated for at least 8 hours at room temperature. Scintillation was measured on Perkin Elmer MicroBeta2 counter.

### Bioluminescence Resonance Energy Transfer (BRET)

48 hours after transfection, HEK293 cells were washed with phosphate buffered saline (PBS), detached with PBS/Trypsin-EDTA (0.25% Trypsin; 1 mM EDTA, Invitrogen), harvested by centrifugation (5 min, 1,000g), resuspended in PBS supplemented with 0.5 mM Ca^2+^ and 0.5 mM Mg^2+,^ and incubated at room temperature on a shaker (app. 250 rpm) until the time of the experiments. The resuspended cells were distributed in 96-well microplates (black/white optiplate, PerkinElmer) and incubated in the presence or absence of ligands. The reading time was 15 min. after agonist addition for dose-response curves.

DeepBlueC coelenterazine (Coelenterazine 400a, Biosynth) was added two seconds before reading using an injector at a final concentration of 5 µM. Measurement of *Renilla* Luciferase (*R*Luc)-mediated luminescence and GFP^2^-mediated emission from each well were performed using a Tecan Infinite F500 microplate reader (Tecan Group Ltd., Männedorf, Switzerland). The BRET2 ratio was determined by calculating the ratio of the light emitted by GFP^2^ (515 nm) over the light emitted by the *R*Luc (410 nm). For BRET1, the ratio was calculated as light emitted by YFP (530 nm) over the light emitted by the *R*Luc (470 nm). The background signal from *R*luc was determined by co-expressing the *R*Luc construct with empty vector, and the BRET1/BRET2 ratio generated from this transfection was subtracted from all other BRET1/BRET2 ratios. Data were analyzed in Graphpad Prism and Excel. Statistical analysis was performed in Excel using Student’s t-test, unpaired, two-tailed.

### Animals

Animal care followed the guidelines of the National Institutes of Health and the experimental protocol was approved by the Danish Animal Experiments Inspectorate. Studies were conducted in male and female C57Bl/6J (WT) mice (Taconic Farms Inc., Denmark). Mice had free access to rodent chow (Altromin, Lage, Germany) and tap water.

### *In vitro* Experiments: Isometric Force Measurements in Mouse Intra-renal Arteries

Intrarenal segmental artery rings were suspended in a Halpern-Mulvany wire myograph (Model 610M, Danish Myo Technology A/S, Aarhus, Denmark) and isometric force development was measured (PowerLab, ADInstruments, Colorado Springs, CO, USA). Two rings per mouse artery were incubated at 37°C in physiological salt solution [in mmol/L: NaCl 115, NaHCO3 25, MgSO4 1.2, K2HPO4 2.5, CaCl2 1.3, glucose 5.5, and HEPES 10 (control solution)] equilibrated with 5% CO2 in air at pH 7.4. Then, the rings were normalized at a resting tension of approximately 13.3 mN and allowed to equilibrate for 30 minutes. Viability of the vascular smooth muscle and endothelial cells was tested by demonstrating contraction to phenylephrine (10–6 mol/L) and relaxation to acetylcholine (10–6 M), respectively.

### Statistical Analysis

All pharmacological data were analyzed using Excel (Microsoft, Redmond, WA) and Prism (GraphPad Software, San Diego, CA); R-SAT data and phosphatidyl inositol hydrolysis data were analyzed using nonlinear regression curve fitting.

## Results

### Integrative Screen for 7TM Receptors Enhancing AT1R Signaling Potency

The R-SAT screen was performed in NIH3T3 cells transiently expressing a β-galactosidase reporter gene as previously reported [21], [26]. Initially, we performed titration experiments to determine the optimal amount plasmid to achieve robust expression of human AT1R for the co-expression analysis, but still not reach the upper limit of response, leaving a window to identify enhancements. We found transfection with 5 ng plasmid-cDNA/well met these criteria, and this amount of plasmid was consequently used in the subsequent screen (data not shown).

We then performed the co-expression R-SAT screen to find receptors with the potential to up-regulate AngII stimulated signaling of AT1R. 123 different 7TM receptors (available 7TM receptors expressed in the pSI vector (Promega) from the ACADIA pharmaceuticals Inc. plasmid database) were co-expressed with AT1R and the effect of co-expression was determined by comparing the AngII dose-response on AT1R expressed alone to AT1R co-expressed with each individual receptor. On each plate AT1R expressed alone was run in parallel to account for any plate-to-plate variation. The data from these experiments are reported in table S1 as fold increase of the EC50 of AT1R plus co-expressed receptor relative to cells expressing AT1R alone.

**Figure 1:**
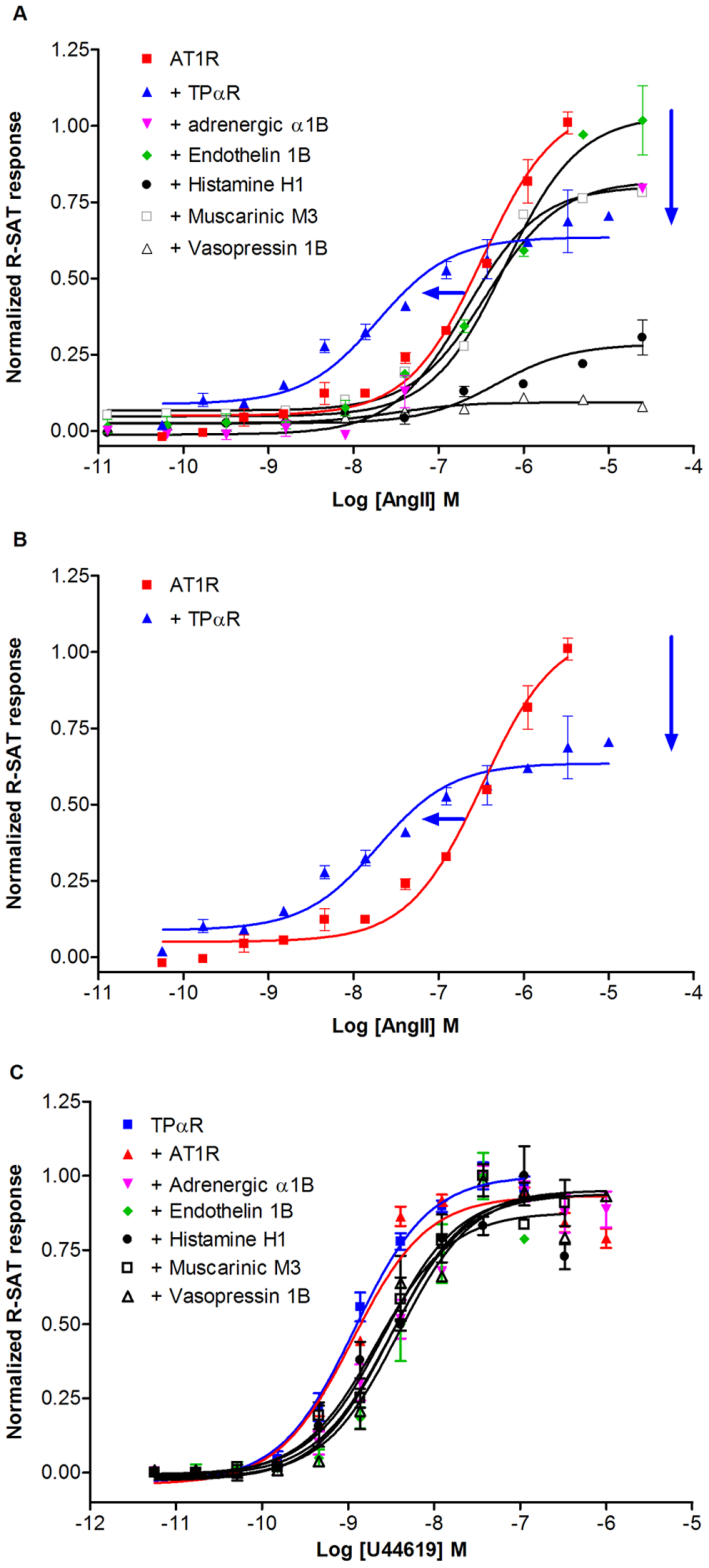
AngII response of co-expression of the AT1R with various 7TM receptors determined by R-SAT assay. AT1R or TPαR were transiently co-expressed in NIH3T3 cells together with of the indicated 7TM receptors and ligand-induced responses determined using R-SAT as described in the methods. Data shown are normalized to the maximal response of AT1R or TPαR alone. **A,** The AT1R was screened against 123 different 7TM receptors, shown are representative dose-response curves after stimulation with AngII for co-expression with TPαR, the Adrenergic α1B, Endothelin 1B, the Histamine H1, the Muscarinic M3, and the Vasopressin V1B receptors. A complete list of data from the screened receptors is reported in table S1. **B**., AngII dose response curve for AT1R expressed alone or co-expressed with TPαR **C**, TPαR agonist response from TPαR co-expressed with the Adrenergic α1B, Endothelin 1B, the Histamine H1, the Muscarinic M3, and the Vasopressin V1B receptors. Average pEC_50_ (±S.D.) values and the number of experiments are reported in Table 1.

The result for a number of representative examples of receptor partners are showed in figure 1a to illustrate the various effects we observed as a consequence of co-expressing different receptors. Interestingly, all but one of the receptors investigated either decreased or did not significantly change the potency of AngII signaling when co-expressed. We chose not to analyze the receptors causing a decrease in AngII response further because it can be a consequence of several different factors, the most likely probably being decreased AT1R-surface expression resulting from nonspecific inhibition of cDNA transcription or AT1R protein translation.

The only receptor significantly enhancing potency of the AT1R response through co-expression was the TPαR. TPαR co-expression results in a significant 11.6 fold potency shift increasing the pEC_50_ value from 6.4 to 7.6 (Fig. 1b). Additionally, the maximal response was lowered by approximately 49%. The mechanism underlying the drop in the maximal response is difficult to address, but it could be a consequence of a decreased AT1R surface expression as discussed above.

Since the TPαR enhanced AngII potency in the presence of the AT1R, we also wanted to know if AT1R co-expression influenced the potency of TPαR agonists as well. To do so, we analyzed the potency with the specific TPαR agonist U46619 in R-SAT in cells expressing the TPαR alone or together with the AT1R. As depicted in Fig. 1c and table 1, AT1R co-expression did not significantly chance the potency of U46619. We also tested how co-expression of five other receptors with the TPαR influenced the potency of U46619, and there were no profound differences in TPαR signaling by co-expression of these receptors either (fig. 1c).

**Table 1:** Pharmacological properties of the TPαR co-expressed with empty vector or various 7TM receptors reported using R-SAT.

Receptor	Additional Receptor/DNA	Drug	pEC_50_	n
**TPαR**				
	pAP4(−)	U44619	8.9±0.1	8
	AT1R	U44619	8.8±0.1	6
	Adrenergic α1B	U44619	8.5±0.2	6
	Endothelin 1B	U44619	8.6±0.2	4
	Histamine H1	U44619	8.4±0.1	6
	Muscarinic M3	U44619	8.6±0.2	6
	Vasopressin 1B	U44619	8.5±0.1	4

NIH3T3 cells were transiently transfected with human TPαR co-expressed with the indicated receptors and stimulated with U44619, a TPαR agonist. R-SAT analysis was performed as described in the materials and methods section. The average pEC_50_ (±S.D.) values and number of experiments are reported.

### Co-expression of TPαR with Various 7TM Receptors Lower Efficacy in R-SAT

**Figure 2:**
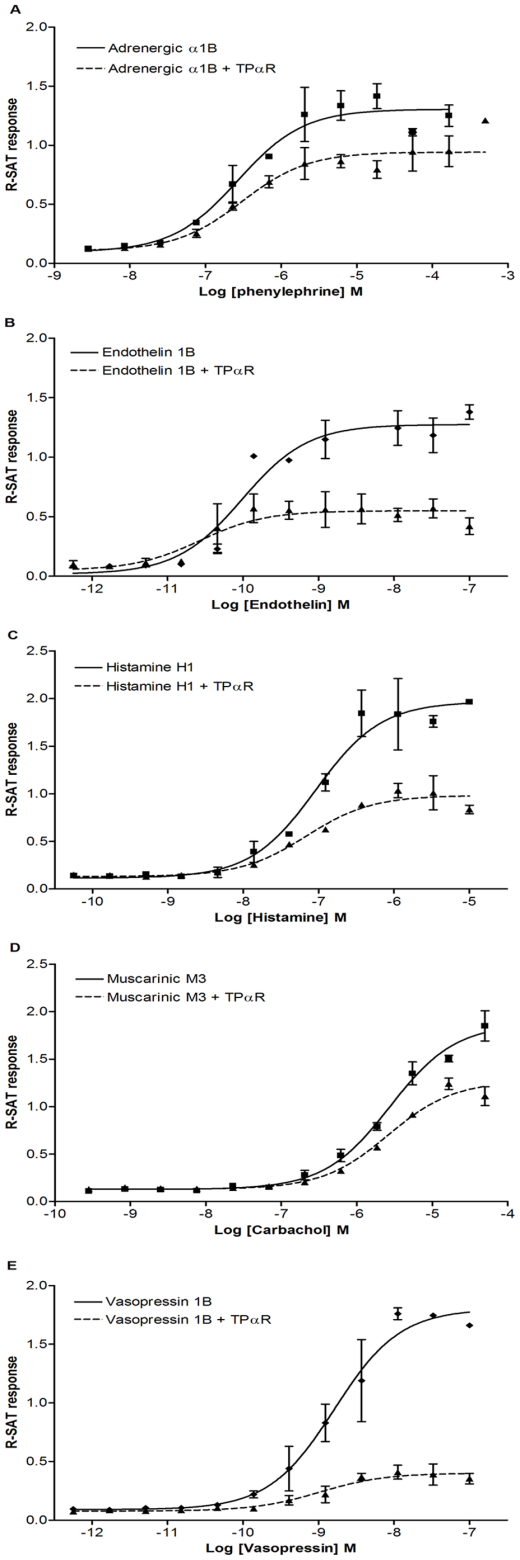
Influence of TPαR on various 7TM receptor signaling in R-SAT. Data shown from representative concentration-response experiments, reported as R-SAT reading. **A,** Adrenergic α1B receptor co-expressed with empty vector or TPαR stimulated with phenylephrine, **B,** Endothelin 1B receptor co-expressed with empty vector or TPαR stimulated with endothelin, **C,** Histamine H1 receptor co-expressed with empty vector or TPαR stimulated with histamine, **D,** Muscarinic M3 receptor co-expressed with empty vector or TPαR stimulated with carbachol, and **E,** Vasopressin 1B co-expressed with empty vector or TPαR stimulated with vasopressin. Average pEC_50_ (±S.D.), and the number of experiments are reported in Table 2.

Next we wanted to study if TPαR potentiates 7TM in general, or if it is specifically linked to the AT1R. To do so, we tested how TPαR co-expression affected the R-SAT response for five 7TM receptors in response to their native ligand (Fig. 2a–e and table 2) For these 7TM receptors, the TPαR promoted a general decrease in efficacy, while pEC_50_ values did not change significantly (table 2). This indicates that TPαR does not enhance 7TM receptor signaling in general.

**Table 2:** Influence of TPαR on various 7TM receptor signaling in R-SAT.

Receptor	Additional Receptor/DNA	Drug	pEC_50_	Max response	n
**Adrenergic α1B**					
	pAP4(−)	Phenylephrine	6.0±0.4	1.3±0.1	5
	TPαR	Phenylephrine	6.0±0.2	0.9±0.1	4
**Endothelin 1B**					
	pAP4(−)	Endothelin	10.2±0.1	1.3±0.1	5
	TPαR	Endothelin	10.4±0.2	0.6±0.1	3
**Histamine H1**					
	pAP4(−)	Histamine	6.9±0.5	2.0±0.1	10
	TPαR	Histamine	6.9±0.5	1.0±0.1	7
**Muscarinic M3**					
	pAP4(−)	Carbachol	5.5±0.2	1.9±0.1	7
	TPαR	Carbachol	5.6±0.2	1.3±0.1	6
**Vasopressin 1B**					
	pAP4(−)	Vasopressin	8.7±0.3	1.8±0.1	3
	TPαR	Vasopressin	9.1±0.4	0.4±0.0	3

R-SAT measured TPαR transfected cells co-expressing: **(a)** Adrenergic α1B receptor stimulated with phenylephrine, **(b)** Endothelin 1B receptor stimulated with endothelin, **(c)** Histamine H1 receptor with histamine, **(d)** Muscarinic M3 receptor stimulated with carbachol, and **(e)** Vasopressin 1B stimulated with vasopressin. The R-SAT analysis was performed as described in the materials and methods section. The average pEC_50_ (±S.D.) values and number of experiments are reported.

### TPαR Induced Enhancement of the AT1R Response in R-SAT is Caused by Paracrine Transactivation of the TPαR

**Figure 3:**
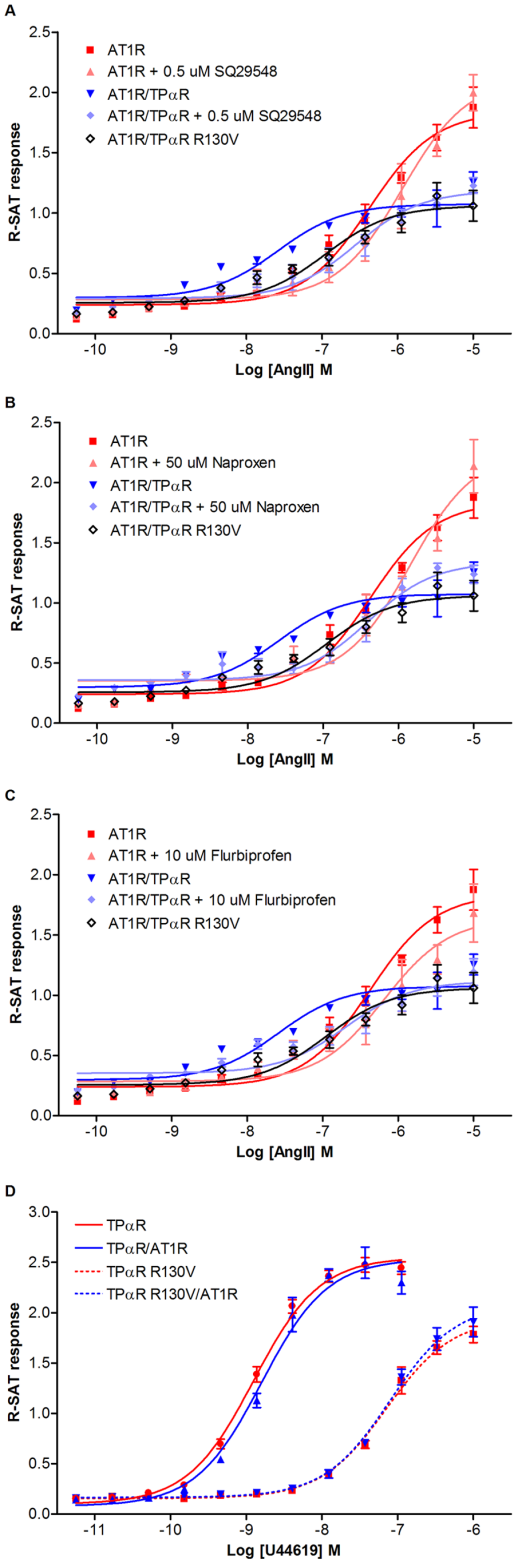
Pharmacological properties of TPαR inhibitors in R-SAT. NIH3T3 cells were transiently transfected with human AT1R alone or co-expressed with the TPαR or the mutant TPαR R130V. R-SAT analysis was performed as described in the materials and methods section. The NIH3T3 cells were stimulated with AngII in absence or presence of **A,** 0.5 µM SQ29548, **B,** 50 µM Naproxen, or **C,** 10 µM Flurbiprofen. **D,** The TPαR R130V receptor was expressed alone or in combination with the AT1R and stimulated with the TPαR agonist, U46619, also the TPαR was expressed alone or in combination with AT1R and stimulated with the U46619. Data shown are from representative concentration–response experiments. Average pEC_50_ (±S.D.) values and the number of experiments are reported in Table 3.

To test if paracrine transactivation of the TPαR due to AT1R mediated TXA_2_ release caused the increase in AngII potency at AT1R’s, we applied SQ29548, Naproxen, and Flurbiprofen in combination with AngII (Fig. 3a–c and table 3). SQ29548 is a highly selective TPαR antagonist [33], while both Naproxen and Flurbiprofen are non-selective COX inhibitors that work by inhibiting both the COX-1 and COX-2 enzymes responsible the synthesis of the TP receptor agonist TXA_2_ [34].

**Table 3:** Pharmacological properties of TPαR inhibitors and TPαR agonist in R-SAT.

Receptor	Additional Receptor/DNA	Drug	Inhibitor	pEC_50_	n
**AT1R**					
	pAP4(-)	AngII		6.4±0.1	4
	pAP4(-)	AngII	SQ29548	5.9±0.1	4
	pAP4(-)	AngII	Naproxen	5.8±0.1	4
	pAP4(-)	AngII	Flurbiprofen	6.2±0.2	4
	TPαR	AngII		7.6±0.1	4
	TPαR	AngII	SQ29548	6.5±0.1	4
	TPαR	AngII	Naproxen	6.4±0.1	4
	TPαR	AngII	Flurbiprofen	6.7±0.2	4
	TPαR R130V	AngII		7.0±0.1	4
**TPαR**					
	pAP4(-)	U46619		8.9±0.0	4
	AT1R	U46619		8.8±0.1	4
**TPαR R130V**					
	pAP4(-)	U46619		7.1±0.1	4
	AT1R	U46619		7.1±0.5	4

NIH3T3 cells transiently transfected with human AT1R in combination with TPαR, the TPαR R130V, or empty vector and stimulated with AngII alone or in presence of TPαR inhibitors SQ29548, Naproxen, or Flurbiprofen. Also, TPαR in combination with either AT1R or empty vector stimulated with U46619, and the mutant TPαR130V in combination with AT1R or empty vector stimulated with U46619 are shown. The R-SAT analysis was performed as described in the materials and methods section. Data represent the mean ± S.D of 4 independent experiments each performed in duplicate.

When TPαR and AT1R where co-expressed all three blockers caused a significant decrease in potency for AngII. Without any inhibitors present, the pEC_50_ value of AngII was 7.6±0.1. But in the presence of inhibitors the pEC_50_ values dropped to 6.5±0.1 for SQ29548, 6.4±0.1 for Naproxen, and 6.7±0.2 for Flurbiprofen, respectively (Fig. 3a–c and table 3). In comparison, the inhibitors only had a weak reduction of AT1R response when expressed alone. When the AT1R was expressed alone the pEC_50_ for AngII curve was 6.4±0.1 without any inhibitors present. In the presence of inhibitors pEC_50_ was slightly reduced; for SQ29548 to 5.9±0.1, Naproxen to 5.8±0.1, and Flurbiprofen to 6.2±0.2. This suggest, that the enhancement in AT1R response in the presence of TPαR might be caused by a long-term paracrine release of TPαR agonist in R-SAT, which can be inhibited by the presence of TPαR antagonist or TPαR ligand synthesis inhibitors.

The loss-of-function mutant TPαR R130V, has a mutation causing G protein uncoupling. The mutation is situated in the conserved E/DRY motif located at the boundary between transmembrane domain 3 and the second intracellular loop [35], [36]. A previous study has shown that the R130V mutant is expressed at similar level as the TPαR wild type [35] and we have tested the expression of the luciferase tagged TPαR wild type and R130V mutant receptor using luciferase measurement, where we find that the luciferase tagged R130V mutant expressed 123% ±4% of the wild type luciferase tagged TPαR (data not shown). Accordingly, the R130V mutant receptor can be used to decipher the importance of receptor expression vs. signaling activity for the gain-of-function event. As depicted in figure 3d and table 3 this mutant has a significantly decreased response in R-SAT. The mutant was used to test if mere presence of TPαR is sufficient to potentiate AT1R-signaling. As depicted in fig 3a-d co-expression of TPαR R130V instead of wild-type TPαR’s reduced the enhancement of AngII potency. These results indicate that presence of fully active TPαR, is necessary to promote a full potentiation of AngII-mediated AT1R response.

### TPαR does not Influence AT1R-mediated Signal Transduction in the “Short Term” Assays

**Figure 4:**
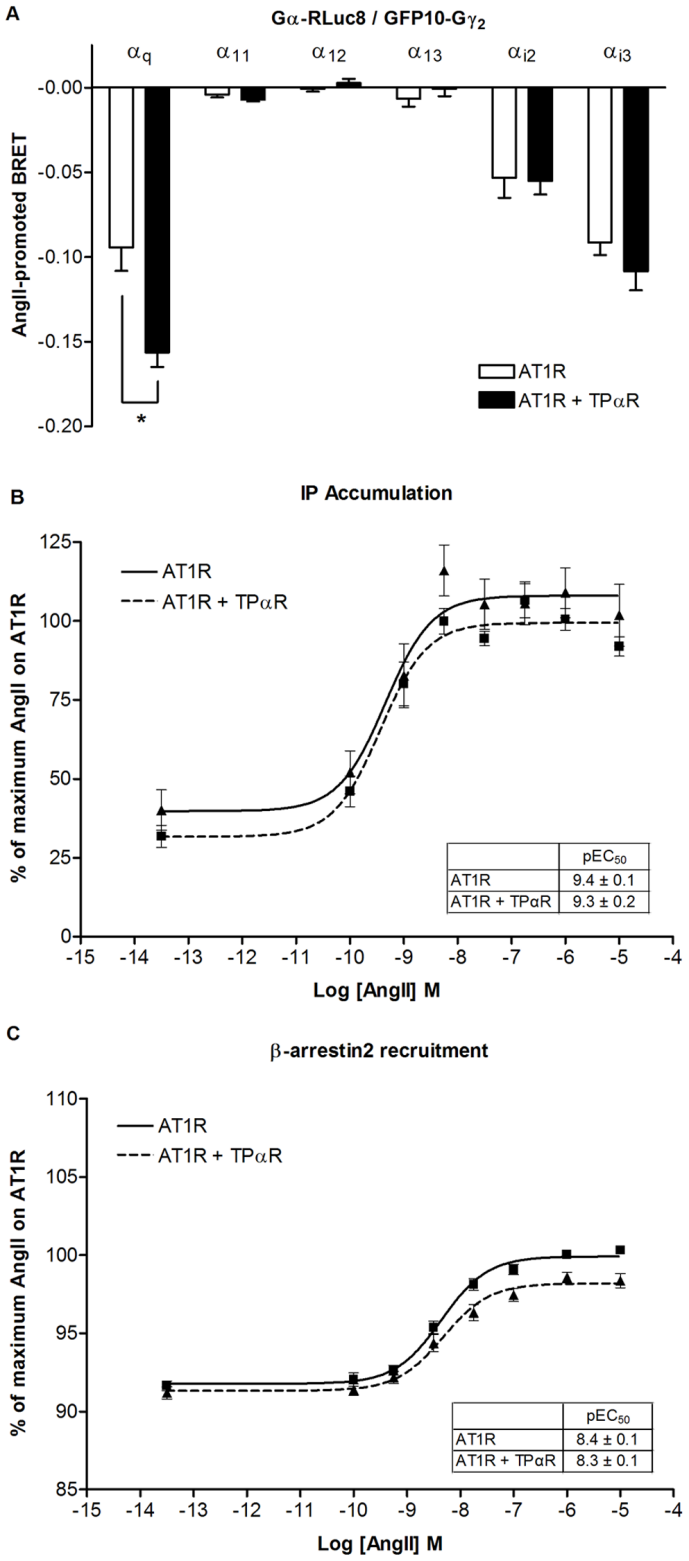
Influence of TPαR on AT1R-mediated signal transduction in the “short term” assays. **A**, BRET2 measured in HEK293 cells co-expressing the indicated Gα subunit tagged with *R*luc8 together with GFP10-Gγ_2_ and Gβ_1_ in the absence of TPαR (grey bars) or in the presence of TPαR (black bars) and stimulated with AngII (1 µM). Results are expressed as the difference in the BRET2 signal measured in the presence and the absence of agonist. Data represent the mean ± S.E.M. of at least 3 independent experiments. * indicates significant difference (P<0.05) as determined by Student’s t test. **B**, Concentration-response curves for AngII-induced IP accumulation in HEK293 cells are depicted as average curves (±95% confidence intervals) from at least three independent experiments performed in triplicate. Data are normalized to percentage of maximum AngII on the AT1R alone. AT1R (2 µg) was expressed alone or co-expressed with TPαR cDNA (2 µg) together with empty vector to reach equal amounts of cDNA in all transfections. **C**, Concentration-response curves for AngII-induced BRET1 measured in real time in HEK293 cells co-expressing AT1R-*R*luc and YFP-β-arrestin2 in absence or presence of TPαR. Curves are depicted as average curves (±95% confidence intervals) from at least three independent experiments performed in duplicate. Data are normalized to percentage of maximum AngII on the AT1R alone.

**Figure 5:**
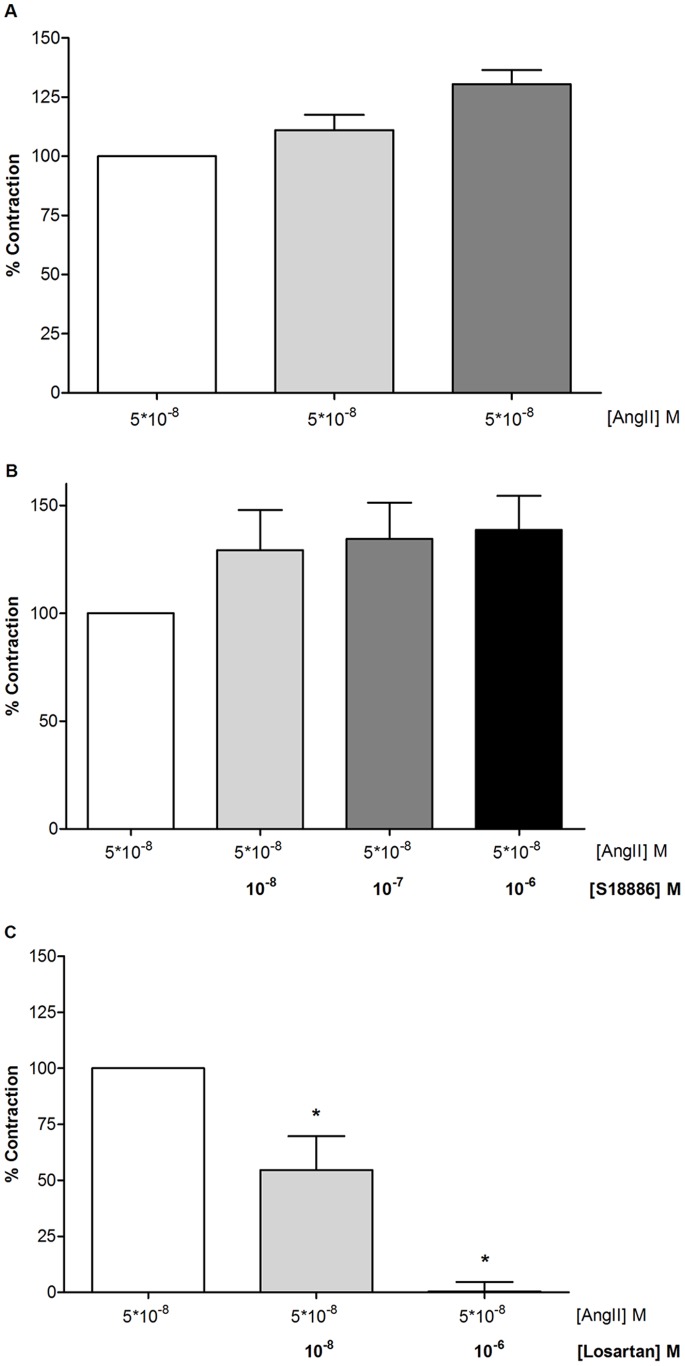
Effect of administration of AngII to mouse intra-renal arteries. **A**, Effect of three consecutive applications of AngII (AngII; 5*10–8 M). **B**, The experiment was repeated in the presence of S18886 (10–8–10–6 M), and **C**, in the presence of losartan (10–8–10–6 M). Data are expressed as percentage of the contraction to AngII (100%) and shown as means ± S.E.M. Asterisks indicate a statistically significant inhibitory effect (P < 0.05, n = 4).

To test if the TPαR had any direct effects on short term AngII responses of the AT1R, we first analyzed the TPαR effects on AT1R on the level of the individual G protein subunits. To estimate the receptor-mediated Gα subunit activation in real time in living cells, we used a BRET assay described by Gales et al. [30], [37]. When the G protein is activated a greater separation between the *R*luc8-tagged Gα_q_ helical domain and the GFP10-tagged Gγ_2_ N-terminus occurs during GDP/GTP exchange [30], [37]. This translates into a decrease in BRET signal following receptor activation. Therefore, this BRET assay allows to us measure conformational changes in the heterotrimeric Gαβγ subunits, which can indicate activation of the G protein. The BRET probe-fusion of either Gα_q_, Gα_11_, Gα_12_, Gα_13_, Gα_i2,_ or Gα_i3_ together with Gγ_2_ were co-expressed together with the complementary Gβ_1_ subunit and the untagged AT1R without or with the TPαR. For most G proteins we did not observe any effect the TPαR on AT1R induced responses. The only difference observed was on Gαq rearrangement. Stimulation with AngII resulted in a robust ligand-promoted decrease in the BRET signal for the probe for Gα_q_ on –0.094±0.014 when AT1R was expressed alone (Fig. 4a). Co-expression with the untagged TPαR decreased the ligand-promoted Gα_q_ BRET signal to -0.156±0.009, indicating that the TPαR increases the Gα_q_ rearrangement resulting from AngII stimulation. AngII-stimulation on the BRET probes for Gα_11_, Gα_12_, Gα_13_, Gα_i2_, or Gα_i3_ did not significantly change promoted BRET signal when TPαR was co-expressed together with AT1R (Fig. 4a). However, as seen in figure 5b, the observed increase in Gα_q_ conformational changes in presence of TPαR did not translate into increased IP accumulation. After stimulation with AngII, similar potencies were observed for the AT1R alone and in combination with TPαR (Fig. 4b). Likewise, the TPαR did not change the AT1R’s ability to recruit β-arrestin. Real time BRET^1^ monitoring of the interaction between AT1R-*R*luc8 and YFP-β-arrestin2 revealed that TPαR does not influence the AngII-mediated BRET signal, as AngII-stimulated AT1R-*R*luc8 recruits YFP-β-arrestin2 with similar potency as co-expression of untagged TPαR (Fig. 4c).

### TPαR Inhibitor does not Influence the Acute AngII-stimulated Contraction in Intra-renal Arteries from Mice

Several studies suggest a functional relation between the AT1R and TPαR *in vivo* and furthermore it has been shown that TPαR inhibitors can suppress AngII-mediated responses [38], [39]. These studies suggest that this effect is most likely through regulation of arterial constriction. To test if TPαR influences AT1R in arteries directly through a short term ligand release, we applied the TPαR inhibitor S18886 on AngII-stimulated arterial contraction in mice (Fig. 5a–c). AngII concentration-dependently contracted blood vessels with a pEC_50_ value of 7.2 (data not shown). Three consecutive application of AngII led to a significant contraction with no difference between the first, second and third administration (Fig. 5a). Inhibition of TP receptors using S18886 had no significant effect on the AngII induced contraction (Fig. 5b). The contractions were significantly inhibited by losartan (Fig. 5c), which shows that TPαR does not influence AT1R-mediated intra-renal arterial contraction in mice in short term studies.

## Discussion

### Signaling Synergism between 7TM Receptors is a Rare Event in the R-SAT Assay

There are many examples in the literature of hetero-dimerization and functional cross-talk between 7TM receptors and therefore it might be expected to be a very common phenomenon [4], [40], [41]. Here, we attempted to analyze the frequency of “functional enhancement” for a particular receptor using the AT1R as an example. This is the first time a comprehensive and systematic investigation of the universality of functional cross-talk between 7TM receptors has been performed. The screen revealed that a number of 7TM receptors had an effect on AT1R signaling. While many 7TM receptors decreased AT1R signaling in the R-SAT assay, the TPαR was the only receptor amongst the 123 receptors we tested that significantly enhanced AT1R signaling (Fig. 1 and table S1). This indicates that functional synergism/potentiation between 7TM receptors is not a promiscuous event but actually requires specific conditions to occur.

The screen was performed in the R-SAT assay. This assay incorporates the combined signaling of multiple signal transduction pathways into a single homogeneous output [31]. The compatibility of R-SAT with receptors of all signaling classes, together with the simple assay format is advantageous when performing large scale screening. Previously, we have used R-SAT successfully to determine the pharmacological properties of a battery of AngII analogs, where some demonstrated increased potencies to AngII [26]. In addition, we previously used the R-SAT assay to identify a gain-of-function for the heterodimerization pair of the GABA_B1_ and GABA_B2_ receptors [21]. In this paper we showed that when we stimulate with the GABA ligand Baclofen on GABA_B1_ or GABA_B2_ receptors when expressed individually, it does not increase the R-SAT response. However, when the two receptor subunits are co-expressed, the R-SAT response shows a robust increase in signaling. This validates that the R-SAT assay can pick up heterodimerization signaling for heterodimerization pairs. Although the assay has proven useful for studying gain-of-function events for AT1R, there are limitations. First; R-SAT does not differentiate between the specific signaling pathways involved, which means that if some receptors enhance certain AT1R signaling pathways and diminish others, the net effect might be an unaltered (or even decreased) R-SAT response. Moreover, the co-expressed receptors could enhance AT1R signaling pathways not detected by R-SAT. Secondly, it is very difficult to quantify AT1R surface expression since the receptors are transiently expressed, and their expression will change over the 6-day time course of the assay. Since co-expression of certain other receptors may decrease surface expression of the AT1R, we may have missed some gain-of-function events. In addition, differences in expression levels between the co-expressed 7TM receptors, will affect the results even though they are expressed in the same vector, they will not express at identical. Thirdly, we only performed the screen at one cDNA concentration, therefore it is possible that we could have picked up more gain-of-function events using different cDNA concentrations.

The screen also revealed a number of receptors that downregulate AT1R signaling. Based on the methodological limitations of the R-SAT assay (see above), it can be difficult to determine whether the observed downregulation of AT1R signaling is caused by a true functional receptor interaction or by a non-specific effect on cell surface expression or by transcriptional/translational quenching. However, downregulation of AT1R signaling by certain 7TM receptors like the Vasopressin 1B and Histamine H1 could be specific for the AT1R (Fig. 1a), since this downregulation was not detected when the two receptors were co-expressed with the TPαR (Fig. 1c). Hence, the screen contains a large number of 7TM receptor interaction data, which would be interesting to further explore in the future.

Nevertheless, we were able to reproduce a number of earlier findings demonstrating the usefulness of the approach. Firstly, a physical interaction between the Bradykinin B_2_ receptor and AT1R has been proposed [20], but has later been disputed by several groups using other assays [21]. As we have previously published, co-expression of Bradykinin B_2_ receptor did not result in any increase in AngII-mediated AT1R response in this screen either (table S1) [21], [22]. Secondly, AT2R is reported to inhibit AT1R signaling [15]. We observed a 6.8 fold decrease in AT1R signaling when AT2R was co-expressed with AT1R (table S1), which is in agreement with that study. Thirdly, in a study in human RPT cells it was shown that the D5, but not the D1 receptor decreases AT1R expression [25]. Consistent with that earlier study, the Dopamine D5 receptor reduced AngII-stimulated response in R-SAT.

### AT1R Activation Most Likely Mediates TXA_2_ Synthesis, which Leads to Paracrine TPαR Activation

As discussed, TPαR was the only 7TM receptor that significantly potentiated AT1R activation. TPαR and AT1R are expressed together in many different cell types and tissues [42], [43], and they have been shown to interact both *in vitro* and *in vivo*, which makes the interaction interesting from a physiological and pharmacological perspective.

Our R-SAT experiments suggest that long term AT1R activation mediates TPαR ligand synthesis leading to paracrine TPαR activation, which then results in an enhanced sensitivity to AngII. 1) The AngII-mediated response on the co-expression of AT1R and TPαR was completely abolished by the co-stimulation with either TPαR antagonist SQ29548 (Fig 2a) or COX inhibitors responsible for TPαR ligand synthesis (Fig. 2b–c). Although, we have not established the expression of thromboxane synthase that generates TXA2, several studies have established that NIH3T3 cells express all the necessary components for activating the TPαR. This includes arachidonic acid [44] and COX-1/2 [45], [46] that are responsible for generating prostaglandin H2 (that in itself can work as an agonist on the TP receptor) [47], [48]. 2) Co-expression of AT1R with a mutant TPαR R130V, deficient in G protein coupling, enhanced the potency of AngII signaling to a much lesser degree than did the wild-type TPαR (Fig. 2d).

On the other hand, the TPαR expression did not affect AT1R signaling in the short term assays**.** In the G protein BRET assay we did observe a change in the AngII induced BRET signal when the TPαR was present (Fig. 4a). However, most G protein responses were unaffected and the increased Gα_q_ rearrangement did not translate into increased IP accumulation, which is the usual outcome of Gα_q_ activation (Fig. 4b). One possible explanation could be that the G protein rearrangement observed in the BRET assay does not represent the canonical active conformation that results in accompanying Gα_q_ mediated phosphatidylinositol production. In addition, the presence of TPαR did not affect the AT1R mediated β-arrestin recruitment (Fig. 4c), and the TPαR inhibitor does not influence the acute AngII-stimulated contraction in intra-renal arteries from mice (Fig 5b). Taken together, this data suggest that long term AT1R activation mediates TXA_2_ synthesis, which leads to paracrine TPαR activation in R-SAT assay whereas that does not occur in the short term assays we have tested. A recent study confirms the lack of acute vascular effect by AngII in TPR knockout vascular smooth muscle cells [49].

The functional relation between that AT1R and TPαR and the very complex and has not yet been fully elucidated. But it is well established, that the TPαR signalling is partly responsible for the development of AngII-mediated hypertension [38], [50]. These studies also suggest that AT1R activation leads to TXA_2_ release followed by a paracrine TPαR activation. 1) Castillo-Hernandez et al. showed that the inotropic and vasoconstrictor effects by intracoronary AngII in hearts from Wistar rats are blocked by COX inhibitors and a competitive antagonist of TPαR, and the vasoconstriction effects by AngII were mimicked by infusion of U46619 [51]. 2) TPαR inhibitors reduced blood pressure in 2K1C Glodblatt hypertensive rats [38]. 3) Francois et al. observed a blunted pressure response in TPαR knockout (TPαR ^−/−^) mice compared to wild type during chronic AngII infusion [50].

### Conclusion

We have performed a large functional screen to analyze for gain-of-function signaling between 7TM receptors, using the AT1R as a model receptor and looked at the ability of different 7TM receptors to enhance AngII-mediated AT1R responses in the R-SAT assay.

Surprisingly, our screen identified the TPαR as the only receptor that significantly potentiated the AngII response. While the screen identified a number of 7TM receptors that are able to decrease AT1R signaling, we only found one receptor that significantly enhanced AT1R potency. Our results suggest that functional enhancement through 7TM receptor cross-talk is a rare event that may require special conditions to arise. The functional relation between that AT1R and TPαR are very complex and has not yet been fully elucidated. Our data suggests that a long-term AT1R activation leads to a paracrine release of TXA_2_ which then activates the TPαR signaling.

Cross-talk between 7TM receptors is an important aspect of 7TM receptor signaling and may have an important influence on the biological output. Even though our results indicate that cross-talk is not a common phenomenon, the functional interaction between physiological relevant receptors has to be accounted for in modern drug development.

## Supporting Information

Table S1 Pharmacological properties of AngII stimulation for the AT1R co-expressed with various 7TM receptors using R-SAT. NIH3T3 cells were transiently transfected with human AT1R alone or co-expressed with various 7TM receptors and the R-SAT analysis was performed as described in the materials and methods section. Fold increase in EC50 for the co-expression of various 7TM receptors together with the AT1R compared to the EC50 value for AT1R expressed alone when stimulated with AngII in each experiment are reported.
(DOC)
