# Supplementary material for: Functional Enhancement of AT1R Potency in the Presence of the TPαR Is Revealed by a Comprehensive 7TM Receptor Co-Expression Screen
Source: PLoS One. 2013 Mar 14;8(3):e58890. doi: 10.1371/journal.pone.0058890 (PMC3597553; doi:10.1371/journal.pone.0058890)
Supplement: Table S1 — Pharmacological properties of AngII stimulation for the AT1R co-expressed with various 7TM receptors using R-SAT. NIH3T3 cells were transiently transfected with human AT1R alone or co-expressed with various 7TM receptors and the R-SAT analysis was performed as described in the materials and methods section. Fold increase in EC50 for the co-expression of various 7TM receptors together with the AT1R compared to the EC50 value for AT1R expressed alone when stimulated with AngII in each experiment are reported. (DOC) [file pone.0058890.s001.doc]

**Supporting Information**

**Table S1: Pharmacological properties of AngII stimulation for the AT1R co-expressed with various 7TM receptors using R-SAT.**

| **Name** | **Subtype** | **Fold increase** | **n** |
| --- | --- | --- | --- |
| Prostanoid | TPα | 11,6 | 10 |
| Dopamine | D4 | 3,3 | 3 |
| Glycoprotein Hormone | FSH | 3,0 | 3 |
| Histamine | H3 isoform II | 3,0 | 2 |
| Calcium-sensing receptor | CaSR | 3,0 | 2 |
| Platelet-activating Factor | PAF | 2,8 | 3 |
| Metastin | GPR54 | 2,6 | 2 |
| Somatostatin | SST3 | 2,2 | 2 |
| Lysophospholipid | EDG2B | 2,0 | 3 |
| Vasopressin | V2 | 1,9 | 2 |
| Prostanoid | EP1 | 1,9 | 2 |
| Parathyroid hormone receptor | PTHR2 | 1,7 | 2 |
| Lysophospholipid | EDG4 | 1,7 | 3 |
| Muscarinic | m1 | 1,7 | 2 |
| Vasopressin | V1B | 1,6 | 2 |
| Prolactin-releasing hormone | PRLRP | 1,5 | 2 |
| Cannabinoid | CB2 | 1,5 | 2 |
| Purinergic | P2Y4 | 1,5 | 3 |
| Urotensin | UTS2 | 1,4 | 2 |
| Melatonin | MT2 | 1,3 | 2 |
| Prostanoid | EP4 | 1,3 | 2 |
| Histamine | H3 isoform I | 1,2 | 2 |
| Serotonin | 5HT1B | 1,2 | 2 |
| Opioid | μ | 1,2 | 2 |
| Adrenergic | β1 | 1,2 | 2 |
| Serotonin | 5HT2B | 1,2 | 3 |
| Serotonin | 5HT2A | 1,2 | 2 |
| Purinergic | P2Y12 | 1,1 | 2 |
| Somatostatin | SST4 | 1,1 | 2 |
| Purinergic | P2Y2 | 1,1 | 2 |
| Bradykinin | B1 | 1,1 | 4 |
| Histamine | H1 | 1,1 | 2 |
| Serotonin | 5HT4A | 1,1 | 2 |
| Purinergic | P2Y11 | 1,1 | 2 |
| Neuromedin U | NMUR2 | 1,1 | 2 |
| Bradykinin | B2 | 1,1 | 2 |
| Prostanoid | IP | 1,0 | 2 |
| Leukotriene | LTC4 | 1,0 | 3 |
| neurotrophin | TRKC | 1,0 | 2 |
| Dopamine | D3 | 1,0 | 3 |
| Bombesin | BB1 | 1,0 | 2 |
| V5 | V5 | 1,0 | 2 |
| Cannabinoid | CB1 | 1,0 | 2 |
| Melatonin | MT1 | 1,0 | 2 |
| Tachykinin | NK2 | 0,9 | 2 |
| Adrenergic | α1a/D | 0,9 | 2 |
| Adrenergic | α2a | 0,9 | 4 |
| Chemokine | CXCR6 | 0,9 | 3 |
| Neuropeptide Y | NPY1 | 0,9 | 2 |
| Melanocortin | MC4R | 0,9 | 2 |
| Vasopressin | V1A | 0,8 | 2 |
| Neuropeptide | NPFF2b | 0,8 | 3 |
| Opioid | κ | 0,8 | 2 |
| Chemokine | CCR5 | 0,8 | 2 |
| Adenosine | A 2a | 0,8 | 2 |
| CXCR2 |  | 0,8 | 2 |
| Lysophospholipid | EDG2A | 0,8 | 2 |
| Endothelin | ETA | 0,8 | 3 |
| Adenosine | A 2b | 0,7 | 2 |
| Adrenergic | β2 | 0,7 | 2 |
| Dopamine | D2 | 0,7 | 3 |
| Gastric Inhibitory Polypeptide | GIP | 0,7 | 2 |
| CCR11 1233 |  | 0,7 | 2 |
| Serotonin | 5HT5A | 0,7 | 4 |
| Serotonin | 5HT1F | 0,7 | 2 |
| Purinergic | P2Y6 | 0,7 | 4 |
| Adrenergic | α1A/c | 0,7 | 3 |
| CCR8 675 |  | 0,7 | 2 |
| Adrenergic | α2c | 0,7 | 3 |
| Secretin | SCTR | 0,7 | 2 |
| Lysophospholipid | EDG5 | 0,7 | 4 |
| Calcitonin | Calcitonin | 0,6 | 2 |
| GABA | GABAB2 | 0,6 | 3 |
| Prostanoid | EP3D | 0,6 | 2 |
| Melanocortin | MC3R | 0,6 | 3 |
| Dopamine | D5 | 0,6 | 2 |
| CXCR1 |  | 0,6 | 2 |
| Tachykinin | NK3 | 0,6 | 2 |
| Oxytocin | OT | 0,6 | 3 |
| Opioid | δ | 0,6 | 2 |
| Neuropeptide | NPFF1 | 0,5 | 2 |
| Adrenergic | α1b | 0,5 | 3 |
| Endothelin | ETB | 0,5 | 3 |
| Serotonin | 5HT1E | 0,5 | 2 |
| Glucagon-like Peptide | GLP1 | 0,5 | 2 |
| Adrenergic | α2b | 0,5 | 3 |
| Cholecystokinin | CCKA | 0,5 | 3 |
| Galanin | GAL2 | 0,5 | 2 |
| Glucagon-like peptide | GLP2 | 0,5 | 2 |
| Growth Hormone Releasing Hormone | GHRH | 0,5 | 3 |
| CCR2A 1278 |  | 0,4 | 2 |
| GABA | GABAB1a | 0,4 | 3 |
| Glucagon | GCG | 0,4 | 3 |
| Histamine | H2 | 0,4 | 2 |
| Parathyroid hormone receptor | PTHR1 | 0,4 | 2 |
| Adenosine | A 1 | 0,4 | 2 |
| Vasoactive Intestinal Peptide | VIP1 | 0,4 | 2 |
| Gonadotropin Releasing Hormone | GnRH | 0,3 | 2 |
| CCR1 1753 |  | 0,3 | 3 |
| Somatostatin | SST2 | 0,3 | 2 |
| Adenosine | A 3 | 0,3 | 3 |
| Serotonin | 5HT1D | 0,3 | 3 |
| muscarinic | m4 | 0,3 | 4 |
| Leukotriene | LTD4 | 0,3 | 3 |
| Thyrotropin Releasing Hormone | TRH | 0,3 | 3 |
| Adrenergic | β3 | 0,3 | 3 |
| Neuromedin | NMUR1 | 0,2 | 3 |
| muscarinic | m3 | 0,2 | 3 |
| Serotonin | 5HT1A | 0,2 | 3 |
| Neuropeptide Y | NPY4 | 0,2 | 2 |
| GABA | GABAB1b | 0,2 | 2 |
| Vasoactive Intestinal Peptide | VIP2 | 0,2 | 2 |
| Neuropeptide Y | NPY2 | 0,2 | 2 |
| Opioid | ORL1 | 0,1 | 2 |
| CCR6 673 |  | 0,1 | 2 |
| XCR1 526 |  | 0,1 | 2 |
| Cholecystokinin | CCKB | 0,1 | 3 |
| Angiotensin 2 | AT2 | 0,1 | 3 |
| Orexin | Orx2 | 0,1 | 2 |
| CXCR3 671 |  | 0,1 | 2 |
| CCR7 674 |  | 0,1 | 2 |
| Lysophospholipid | EDG1 | 0,1 | 2 |
| Orexin | Orx1 | 0,0 | 2 |

**Table S1:** NIH3T3 cells were transiently transfected with human AT1R alone or co-expressed with various 7TM receptors and the R-SAT analysis was performed as described in the materials and methods section. Fold increase in EC_50_ for the co-expression of various 7TM receptors together with the AT1R compared to the EC_50_ value for AT1R expressed alone when stimulated with AngII in each experiment are reported.
